# Large area few-layer TMD film growths and their applications

**DOI:** 10.1088/2515-7639/ab82b3

**Published:** 2020-04-27

**Authors:** Srinivas V Mandyam, Hyong M Kim, Marija Drndić

**Affiliations:** 1 Department of Physics and Astronomy, University of Pennsylvania, Philadelphia, Pennsylvania 19104, United States of America; 2 Department of Electrical and Systems Engineering, University of Pennsylvania, Philadelphia, Pennsylvania 19104, United States of America

**Keywords:** transition metal dichalcogenides, 2D materials, large area, CVD, PLD, ALD, transistor

## Abstract

Research on 2D materials is one of the core themes of modern condensed matter physics. Prompted by the experimental isolation of graphene, much attention has been given to the unique optical, electronic, and structural properties of these materials. In the past few years, semiconducting transition metal dichalcogenides (TMDs) have attracted increasing interest due to properties such as direct band gaps and intrinsically broken inversion symmetry. Practical utilization of these properties demands large-area synthesis. While films of graphene have been by now synthesized on the order of square meters, analogous achievements are difficult for TMDs given the complexity of their growth kinetics. This article provides an overview of methods used to synthesize films of mono- and few-layer TMDs, comparing spatial and time scales for the different growth strategies. A special emphasis is placed on the unique applications enabled by such large-scale realization, in fields such as electronics and optics.

## 1. Introduction

Transition Metal Dichalcogenides (TMD) are a family of compounds of the general formula MX_2_, where M is a transition metal (Mo, W, etc) and X a chalcogen (S, Se, etc). These materials share many of the properties that make graphene valuable while also gaining a host of novel ones [1]. While graphene is a gapless semimetal, TMDs such as MoS_2_ or WSe_2_ possess a direct band gap in the monolayer form. They can be inherently n- or p- typed, or ambipolar, or even metallic [2]. Like graphene, these materials have excellent transport properties derived from low dimensionality. With the addition of an inherent direct band gap and p- or n- typing, TMDs are uniquely valuable in digital logic and optoelectronics [3]. TMD band structures can be finely tuned by varying layer number or by doping [4]. This allows, for example, the inherent potential of TMDs as efficient photodetectors and light-harvesters to be utilized with great freedom [3, 5–9]; by doping, the band gap of a TMD can be shifted to lie within the band gap range of the solar spectrum [2]. TMDs are mechanically robust, capable of withstanding significant stress and strain [3, 10–13]. This makes TMDs a promising material for use in device fabrication, particularly in view of the current trend of ‘wearable electronics’ [3, 10–13]. TMDs are distinguished among 2D materials by uniquely strong spin orbit coupling, and they can exhibit exotic phenomena such as the valley Hall effect and optical valley Zeeman effect [14, 15]. Stacking TMDs also enables the observation of striking new quantum phenomena, such as modulation of the band structure as a function of twist angle, reminiscent of ‘magic-angle’ graphene [16, 17].

Much of the work on TMDs is somewhat modeled upon previous works on graphene. Graphene was famously first realized by exfoliation of layers of bulk graphite. Initial work on TMDs proceeded similarly, with geologic 3D crystals of the material in question serving as the source [1, 18]. However, exfoliated samples cannot be controllably made large and in a uniform and repeatable way [19]; the few-atom thick nature of TMDs and the relative size of peeling force involved render the peeled atomic layers extremely fragile and highly susceptible to exfoliation in random shapes and sizes [18]. Quality, scalability, and reliability are key for meaningful industrial applications, but also to facilitate faster progress in fundamental studies. More sophisticated methods rely on assembling the material from its constituent atoms, allowing for control on the morphology and quality of the final product [1]. This review will address multiple such methods and describe the challenges of achieving large scale film growth with them.

Much research is performed on so-called ‘flake growths’ of TMDs, wherein flakes of the material on the order of 10–100 microns in diameter are synthesized on a substrate (with high coverage naturally being ideal). While these growths allow exploration of material properties and small-scale device fabrication, they are suboptimal for challenges of industrial relevance. Graphene growths were likewise flake-based early on, but modern methods can reliably grow sheets of the material [2]. This has enabled manifold commercial applications, as well as novel research possibilities. Graphene architectures on the centimeter scale [20] are now possible, allowing interfaces with macroscopic systems such as tissues [21]. This is likewise a natural goal for TMD growths, and this review aims to cover the possibilities enabled by such an advance.

Given the rapid pace of 2D material advancements, this review will center around the best current methods for TMD film growths. Prior results may be found in other reviews [1, 10, 22–25]. While these previous reviews generally highlight film growths as a pleasant byproduct of ongoing synthesis efforts, little coverage is given to the specific difficulties of film synthesis and the new applications made possible by it, in comparison to normal flake growths. The definition of ‘large area’ has changed over the years, in this review we interchangeably use ‘large area’, ‘wafer scale’ and ‘film growth’ to refer to growths on the centimeter scale (as opposed to the 10-micron scale of ‘flakes’). This review will focus on the challenges and unique benefits of TMD film synthesis and offer future directions.

## 2. TMD film synthesis methods

Since ‘top-down’ exfoliation based methods for TMD isolation result in flakes ranging from a few to a hundred microns in size, effective film growth methods must rely on creating the material from its constituents [26]. All such methods, listed below in sections 2.1 to 2.4, rely on transferring atoms from a source to a substrate, where they can combine to produce the substance in question. The identities of the substrate, the source, the transfer method, and the reaction conditions are highly variable between approaches, and serve as the method by which products are finely controlled. The challenge, then, is to pick the correct variables to enable film growth. What follows is an overview of common methods used in the field, focusing on the unique challenges of each and the film growths achieved with them.

### 2.1. Metal organic chemical vapor deposition (MOCVD)

**Figure 1.**
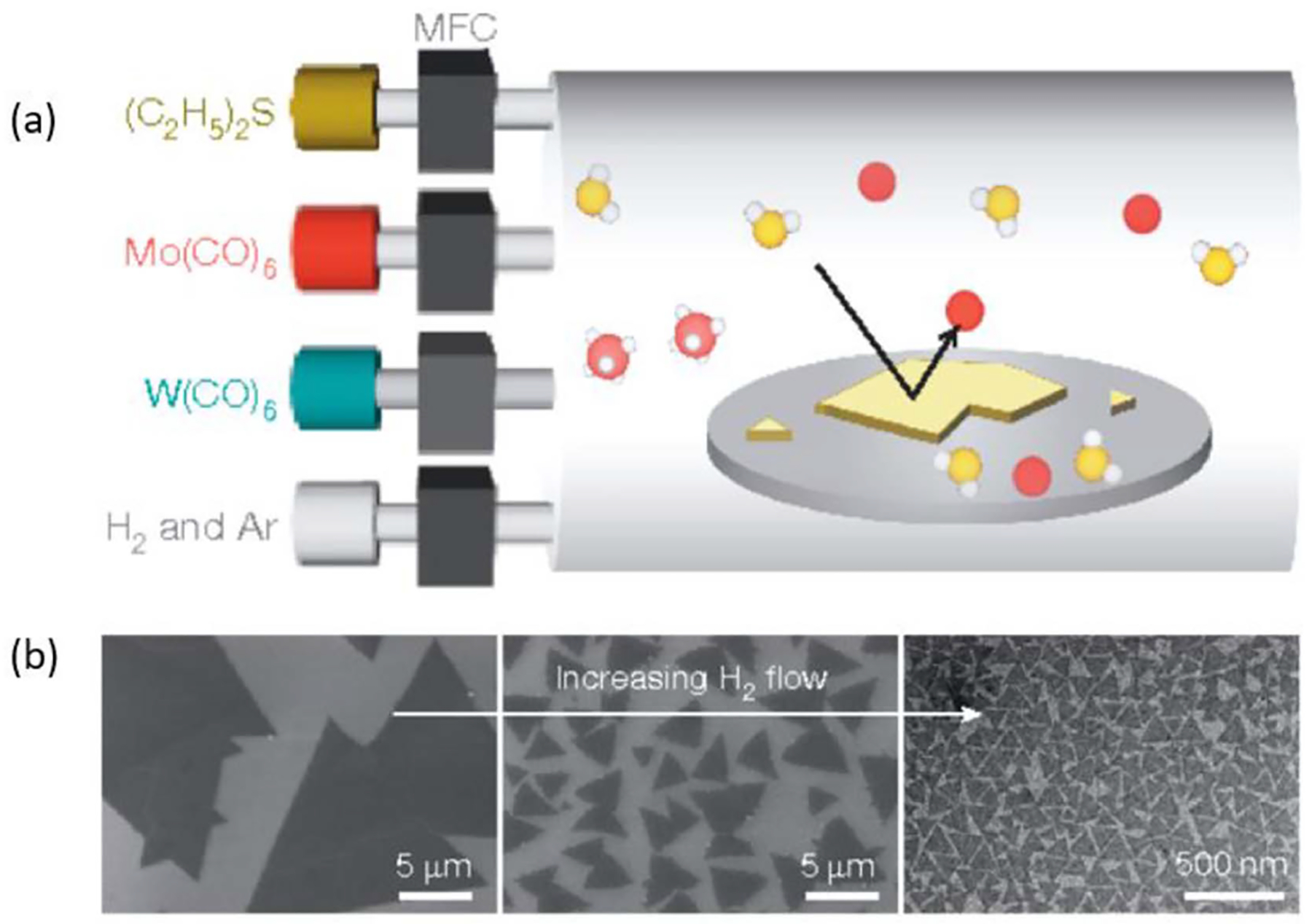
(a) Diagram of MOCVD growth setup. Precursors were introduced to the growth setup with individual mass flow controllers (MFCs). Red, Mo or W atom; yellow, S atom; white, carbonyl or ethyl ligand. (b) Grain size variation of monolayer MoS_2_ depending on the hydrogen flow rate; from left to right, 5 standard cm^3^ min^−1^ (sccm) (SEM image shown), 20 sccm (SEM) and 200 sccm (TEM). Panels adapted with permission from [27]. Copyright 2015 Nature Communications.

This procedure involves reacting gases of pure metalorganic compounds, which contain atoms of the final product bonded to organic compounds. At high reaction temperatures, pyrolysis of these compounds leads to a detachment of the auxiliary organic groups, allowing for the transition metal and chalcogen to react on the substrate (figure 1). Substrates may be crystalline, like sapphire, or amorphous, like SiO_2_. The purity of the reactants allows for high homogeneity of growths, and reactor setups can be made large enough to enable wafer scale growth. This is a reliable method of synthesizing MoS_2_—arguably the TMD that has received the most attention in research thus far—and many groups have been able to synthesize MoS_2_ on the wafer scale through this method. Kang *et al* [27] were able to achieve 10 cm diameter films of monolayer MoS_2_ on a Si/SiO_2_ substrate. The samples were highly uniform and had excellent electronic mobility (114 cm^2^ VsZ^−1^ at 90 K) [27]. This enabled fabrication of 8100 commonly back-gated field effect transistors on a single sample, with 99% yield. This was made possible by advances in MOCVD technology, where fine control of precursor injection allowed for a precise tuning of reactant concentrations. Notably, maintaining a consistent low partial pressure of Mo throughout the growth forces the material to be synthesized layer-by-layer as opposed to a haphazard growth of monolayer and multilayer across the substrate. Unfortunately, low reactant flux results in a slow growth: the full process requires 26 h. Given the energetic costs of the growth process, including the demands of temperatures above 500 °C and pressures below 10 Torr, a shorter growth time would make this strategy far more practical [27]. Kalanyan *et al* [28] developed a novel ‘pulsed’ MOCVD technique, in which injection of precursors were discretized. This method yielded highly uniform 5 cm diameter films of MoS_2_ on Si/SiO_2_ substrates. These growths produced smaller areas than those achieved by Kang *et al*, but instead of requiring 26 h they may be achieved in as little as 90 s [28]. The discretization of precursor injection allowed for precise control of layer number, with each pulse of precursor calibrated to offer around one layer’s worth of reactants. These growths relied on the sublimation of solid metalorganic precursors into the reaction chamber [28]. Choi *et al* [29] called the use of solid precursors into question, noting that it was generally difficult to achieve repeatable growths with them due to variability in sublimation rates. Instead of using multiple separate solid precursors, they chose to use a single liquid precursor containing all reactants [29]. This allowed for a uniform delivery of reactants, with area coverage scaling linearly with growth time and 2 cm-scale monolayer coverage achieved in 15 min. While this growth method lacks in speed and scale compared to the two previously mentioned growths, the use of a single liquid precursor streamlines the process greatly, allowing for a highly repeatable procedure [29].

Efforts to synthesize films of WS_2_ have proceeded alongside MoS_2_ growths. Compared to MoS_2_, WS_2_ is distinguished by superior carrier mobility and a strong photoluminescent response [30]. However, tungsten reacts less readily with chalcogen atoms compared to molybdenum, so in general tungsten TMD growths tend to lag behind their molybdenum counterparts [31]. Using a modified version of their MoS_2_ growth protocol, Kang *et al* [27] were able to achieve 10 cm diameter films of monolayer WS_2_. However, the homogeneity and quality of these films did not compare to the synthesized MoS_2_ films: only 60 good field effect transistors could be fabricated on the whole 10 cm wafer, compared to 8100 for MoS_2_. Choi *et al*’s [29] liquid precursor strategy, when modified for WS_2_, only yielded flakes on the order of 10 microns. MOCVD growth of WS_2_ that is both wafer-scale and high quality still proves elusive.

The selenide TMDs, MoSe_2_ and WSe_2_, have smaller band gaps and even stronger spin–orbit coupling than their sulfide counterparts [2, 5, 23, 32]. Historically, the selenide TMDs have proven more challenging to synthesize owing to Se’s lower reactivity [33]. To our knowledge, no reliable method for wafer scale thin film MoSe_2_ growth via MOCVD exists [25].

WSe_2_, distinguished by intrinsic p-typing and especially strong spin–orbit coupling, is limited to flake growths. Eichfeld *et al* [34] obtained MOCVD flakes of WSe_2_ on the order of 10 microns wide by fine tuning the injection ratio of W and Se atoms. Zhang *et al* [35] observed that MOCVD WSe_2_ growths tended to produce poor quality graphene as a byproduct due to the lingering of carbon atoms from the organometallic precursor, and found that using an inorganic Se precursor helped ensure sample purity.

MOCVD serves as a promising platform for TMD film growth due to its ability to maintain a steady flux of reactants. Uniform delivery is especially important for film growth; uneven growth rates across the substrate lead to a product of inconsistent composition. Since the promise of large-scale TMD architectures relies upon synthesizing homogenous materials, precise control over growth reactions is essential. However, MOCVD reactants, being organic compounds, often form TMD films with significant carbon contamination [35]. Naturally, this severely compromises sample quality. Choi *et al* [29] were able to use water to oxidize carbon out of grown lattices, offering a general method to enhance MOCVD sample purity. Zhang *et al*’s [35] strategy to minimize carbon in precursors also serves as a valid strategy. While MoS_2_ can be reliably realized on the wafer scale, MOCVD methods for other TMDs need to be advanced significantly in both scale, quality, and efficiency.

### 2.2. Atmospheric pressure chemical vapor deposition (APCVD)

While MOCVD is an excellent way to rapidly scale up synthesis, the reactants used in the process tend to be highly toxic [36]. The extremely low pressures and flammable reactants make the growth setup precarious to work with. While this toxicity can be dealt with in an industrial setup, it still represents an additional hassle. APCVD growths operate on the same principle as MOCVD in terms of delivering sublimated material constituents to a growth substrate, but precursors are inorganic and generally much safer. This also eliminates the need for carbon removal. In addition, these growths are conducted at atmospheric pressure which allows for cheaper, simpler setups [19]. Most importantly, MOCVD growths tend to have a high density of grain boundaries, with grain sizes on the order of hundreds of nanometers to a few microns [37]. 2D material films are composed of intersecting flakes. If the lattices of two intersecting flakes are mismatched, this will result in a grain boundary which hinders electronic flow and lowers the chemical and structural quality of the material [37]. Ideal films are composed of fewer, larger flakes meshed together: a high nucleation density increases the probability for grain boundaries. To limit nucleation density, lower fluxes of reactants should be used. However, this will also limit flake size. Consequently, wafer scale growths must strike a delicate balance between grain size and film size [38]. While APCVD tends to be worse at growing large area films, the films tend to have larger grains and be of consequent higher electronic quality.

As with MOCVD, APCVD MoS_2_ growths are most advanced of all TMDs. Ma *et al* [38] achieved 1 cm-scale MoS_2_ growths on a sapphire substrate with a room temperature electronic mobility of 192 cm^2^ Vs^−1^. The key to this relatively high mobility was a low density of grain boundaries; their growth had grain sizes on the order of hundreds of microns. This was achieved by reducing nucleation density via suppressing sulfur flux: instead of using elemental sulfur, MoS_2_ powder was used as the source [38]. Dumcenco *et al* [39] likewise achieved similarly large grain sizes in a 1 cm-scale growth, enabling fabrication of transistor channels up to 80 μm long with mobility that did not fall off with length, a testament to better sample quality. For these growths, a sapphire substrate was used. Being crystalline, sapphire primes flakes to grow in orientations aligning with its own crystal axes, increasing the chances that two intersecting flakes will be aligned. Graphene has been similarly used as a crystalline base for MoS_2_ film growth [40]. However, sapphire is relatively expensive compared to other substrates such as Si/SiO_2_ and cannot be used as a substrate for transistors. With Si/SiO_2_ substrates, electronics can be patterned *in situ*. This avoids a transfer process that may increase the risk of contamination and damage. Of course, an amorphous substrate like SiO_2_ cannot compel flakes to align while forming, increasing grain boundary density and lowering the general quality of the synthesized TMD. Even so, Tao *et al* [41] managed to achieve 1 cm scale films with grains exceeding 100 microns on Si/SiO_2_. This was achieved by occluding the Mo precursor container and tilting the substrate face downstream, limiting reactant flux and consequent nucleation density [41]. Large grains allowed for larger electronic fabrications, as uniformity in material composition could be guaranteed over a larger range. Naturally, these groups must compensate for low flux by tuning other parameters, such as by raising growth time. These growths require around 20 min, comparable to MOCVD efforts [41]. Qian *et al* [37] achieved a significant advance in balancing low nucleation and high growth rates, observing that keeping Mo source temperature low, the substrate temperature high, and the carrier gas flow rate low would allow for grain sizes of 200 microns and films close to 3 cm across. While this growth was not entirely selective for monolayer (having some minor bi, tri, and tetralayered regions), this work shows how MOCVD growth sizes may be approached while maintaining large grains. Another concern is layer number control. TMD band structures change dramatically as a function of layer number, so bilayer and trilayer films gain unique properties that can be exploited [19]. For instance, bilayer TMD films can support interlayer excitons [42]. Jeon *et al* [19] achieved centimeter scale selective growth of both bilayer and trilayer MoS_2_. They found that pre-treating the Si/SiO_2_ substrate with oxygen plasma changed surface chemistry in a way that enabled layer control. Increasing pre-treatment time led to selective increase in layer count. The mechanism for this selectivity is not fully understood, but Jeon *et al* posit that a highly-treated substrate binds reactants tighter, changing the reaction progression [19].

As with MOCVD, WS_2_ films have proven more challenging to synthesize. Zhang *et al* were able to achieve 1 cm-scale monolayer WS_2_ growth on a sapphire substrate by manipulating growth conditions to maintain high growth and low nucleation in a manner similar to Qian *et al*’s efforts [6, 37]. Once again, sapphire aids in the alignment of merging flakes during growth. Lan *et al* [43] also achieved a centimeter-scale monolayer growth on sapphire, seeding the precursor on the substrate was essential to achieve this. Chen *et al* [44] treated Si/SiO_2_ substrates with 3-aminopropyltriethoxysilane (silanization) before coating them with a tungsten oxide containing solution. The silanization treatment enabled a uniform dispersion of tungsten precursor [44]. As with MOCVD, uniformity allows for a homogenous growth, enabling film synthesis. This work resulted in 1 cm-scale growth of monolayer WS_2_ films, with grains on the order of 100 microns [44]. However, the silanization process essentially adds a ‘contaminant’ layer which would interfere with *in situ* device fabrication [44]. Hu *et al* [45] attempted growth directly on a Si/SiO_2_ substrate, achieving flakes 160 *μ*m-wide with morphologies controlled by the ratio of S and W source fluxes. While not a film growth, this serves as a promising first step to growing large area WS_2_ directly on SiO_2_.

One cm-scale MoSe_2_ films were grown by Lu *et al* [33] on Si/SiO_2_. These films were mostly monolayer and bilayer composites. While the grain sizes were small (on the order of hundreds of nanometers), the more daunting selenide growth kinetics make optimizing grain size and growth rate simultaneously difficult [33]. Shim *et al* [46] achieved pure monolayer growths of MoSe_2_ on the scale of hundreds of microns on Si/SiO_2_, sapphire, and graphene; Zhao *et al* [5] managed to selectively grow monolayer films of MoSe_2_ on the millimeter-scale on Si/SiO_2_, with grains on the order of microns. These results were achieved by methods analogous to those used for MoS_2_, using MoO_3_ powder as the molybdenum source and then selenizing them (instead of sulfurizing) with elemental Se [46]. WSe_2_ growths likewise proceed with WO_3_ as the tungsten source instead. Huang *et al* [47] achieved a 1 cm by 2 cm continuous film of monolayer WSe_2_ on sapphire, with domains around 5 microns in width. There has been little progress in creating grains on a size commensurate with those for other TMD growths.

**Figure 2.**
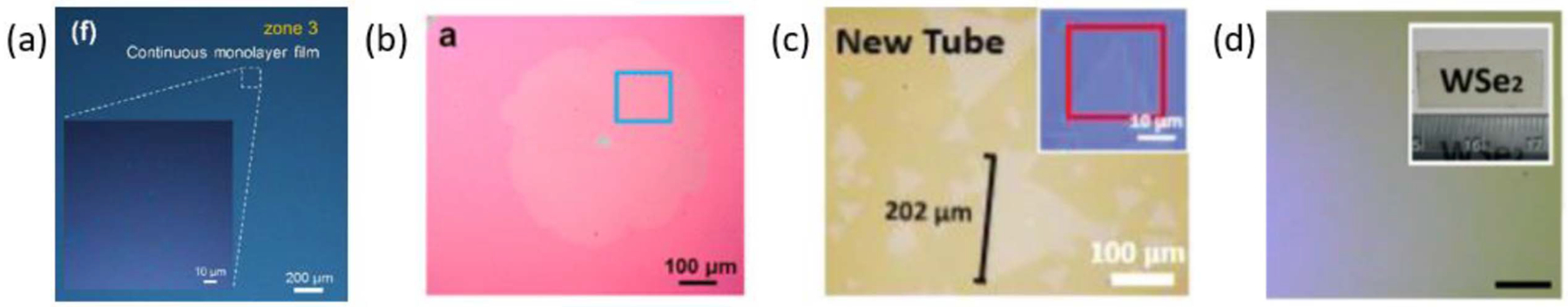
Schematic of films from APCVD (a) Optical images of as-grown MoS_2_. (b) Optical image of the as-grown MoSe_2_ monolayer on SiO_2_/Si substrate. (c) Optical image of monolayer WS_2_ crystals on sapphire grown by a new CVD tube. (d) Optical microscopy images of WSe_2_ film grown at 750 °C. Scale bar is 10 μm in length. Panels adapted with permission from (a) [41], (b) [46], (c) [6], (d) [47]. Copyrights 2017, 2014 American Chemical Society, 2018 Advanced Optical Materials, 2013 American Chemical Society.

While APCVD struggles to grow films on the same scale as MOCVD, the produced material tends to be of higher quality. This is due to the increase in grain size and the use of inorganic precursors, which avoids carbon contamination. The simplicity and nontoxicity of the method makes it a compelling pathway to achieving the industrial synthesis of mono- and few- layer TMD films (figure 2). As with MOCVD however, work must be done to advance the growths of WS_2_, MoSe_2_, and WSe_2_ to the level of MoS_2_, and growths still must be optimized to achieve wafer scale synthesis.

### 2.3. Pulsed laser deposition (PLD)

**Figure 3.**
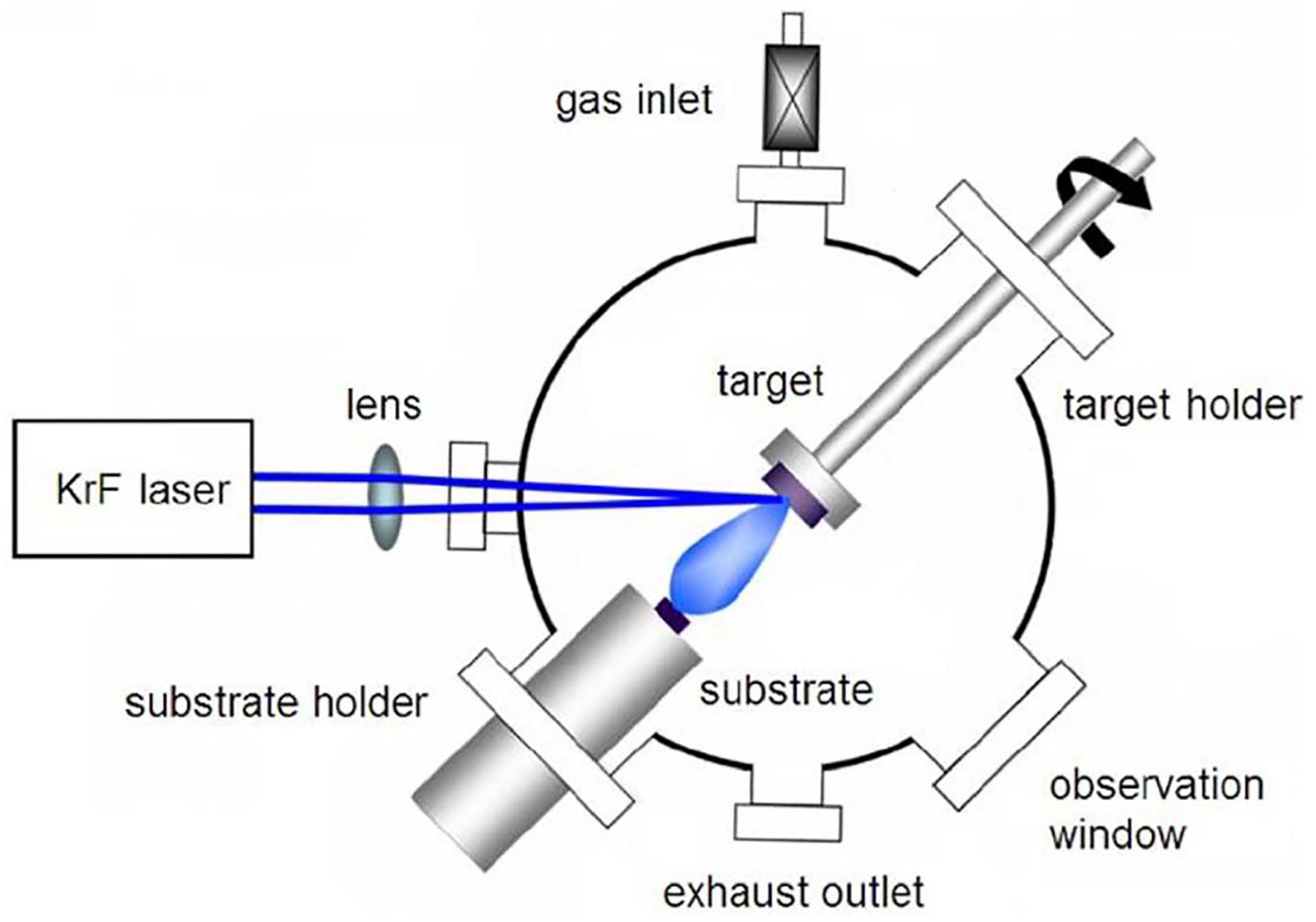
Experimental setup for graphene growth using Pulsed Laser Deposition. Adapted from [48]. Copyright IOP Science 2014.

Pulsed Laser Deposition (PLD) addresses some of the shortcomings of CVD-type TMD growths. It operates on the principle of using a laser to ablate particles from a bulk sample of the desired material, the particles then condense on a substrate under high vacuum (~10^−6^ Torr) to form thin films [48] (figure 3). While CVD growths require reaction temperatures around 800 °C–1000 °C, PLD growths can comfortably occur around 500 °C [3, 7, 49]. In addition, the laser will precisely ablate a certain amount of material, and so can be ‘pulsed’ to achieve fine control on the final layer count of the product. Lastly, the growth speed is limited only by the pulse rate of the laser, permitting faster growths [48]. Siegel *et al* [50] grew 2-cm-scale MoS_2_ on sapphire, observing that the final layer count of the product was a linear function of the number of laser pulses used to ablate the source MoS_2_ powder. In this way, they selectively grew pure monolayer, bilayer, and trilayer films with 50, 100, and 150 pulses respectively [50]. This trend stayed consistent all the way to 3000 pulse growths, which produced 60-layer films. Growths occurred at 700 °C, with a monolayer being synthesized in a relatively quick 5 min [50]. Serna *et al* [49] explored the effect of growth temperature, attempting temperatures from 700 °C down to 300 °C. They observed that the stoichiometric ratio of Mo to S suffered at lower temperatures, with S atoms lacking [49]. They hypothesized that the reaction kinetics at lower temperatures favored S being ablated from the sapphire growth substrate [49]. Even so, large area films formed at temperatures far too low for CVD reactions, hinting that further optimization may make such efficient growths possible. Jiao *et al* [51] attempted MoS_2_ PLD growth on Si/SiO_2_, which enabled FET device fabrication. However, they observed mobilities and on/off ratios (0.124 cm^2^ Vs^−1^ and 500, respectively) several orders of magnitude below those observed in CVD growths [51]. Barvat *et al* [42] observed something similar in their MoS_2_ film growths, and performed further tests to conclude that sulfur deficiencies were a possible culprit. Fominski *et al* [52] attempted to address this stoichiometric defect by immersion of reactants in an atmosphere of low pressure sulfur vapor and hydrogen sulfide. This helped to rectify the S/Mo ratio to around 2.1. However, this process was observed to cause local disturbances to the MoS_2_ crystal structure [52].

WS_2_ growths have proceeded analogously to MoS_2_ growths. Yao *et al* [7] achieved 1-cm-scale growths of multilayered WS_2_ on SiO_2_. Interestingly, this sample had a mobility exceeding that of CVD grown multilayer WS_2_. Possibly, the interior of the multilayered sample was protected from sulfur ablation. Tian *et al* [53] achieved a 1-cm-scale growth of monolayer WS_2_ on sapphire at 500 °C, a temperature even cooler than the one used for their MoS_2_ growth efforts. They observed that film quality improved significantly upon annealing in a sulfur rich environment, suggesting that sulfur vacancies are also a challenge for monolayer WS_2_ [53]. Rathod *et al* [54] further explored the consequences of stoichiometry, growing centimeter-scale monolayer films on Si/SiO_2_ under various laser fluences. Increasing laser fluence tended to decrease sulfur in the final product [54]. They observed that sulfur is expected to preferentially desorb from the substrate under the impact of high reactant flux, so a smaller laser power helped to preserve sulfur in the final product [54]. By sufficiently lowering laser fluence, S/W ratio improved from 1.1 to 1.8 which improved mobility from 0.1 to 6.5 cm^2^ Vs^−1^ [54]. Annealing in a sulfur rich environment served to improve mobility further to 19.5 cm^2^ Vs^−1^ [54]. This shows the crucial role proper stoichiometry plays in material quality.

The reaction kinetics of selenium bring about greater difficulty in the synthesis of selenide TMDs via PLD [55]. To our knowledge, no centimeter-scale PLD growth for MoSe_2_ exists. Even so, Ullah *et al* [55] achieved continuous monolayer MoSe_2_ films on the order of hundreds of microns by utilizing a hybrid process, wherein PLD was used to deposit MoO_3_ on both sapphire and Si/SiO_2_ substrates, which were then selenized in a CVD setup. WSe_2_ PLD growths have shown more promise. Zheng *et al* [3] grew few-layer WSe_2_ on Si/SiO_2_ that was 2 cm in diameter, but with grain sizes on the order of tens of nanometers. Seo *et al* [56] achieved 1-cm-scale selective growths of monolayer, bilayer, and trilayer WSe_2_ on Si/SiO_2_ at 500 °C. As with earlier work on MoS_2_, they observed a direct relationship between laser pulse number and layer count. They noticed a ‘splashing effect’, wherein undesirable reactant nanoparticles attached themselves to the surface of the grown material, Romanov *et al* [46, 57] noticed similar effects during MoSe_2_ PLD growth. Both groups concluded that distancing source and substrate minimized splashing, though too much distance interfered with product formation [56, 57]. Seo *et al* [56] fabricated FETs on the grown WSe_2_ sample, and observed mobilities and on/off current ratios of 0.005 cm^2^ Vs^−1^ and 130, respectively. These compare poorly to equivalent CVD efforts (with both several orders of magnitude lower), and a high density of grain boundaries was suspected as the cause.

The PLD strategy has numerous strengths. It is energy efficient, capable of working at low temperatures. It is quick, with most growths taking on the order of a few minutes. Most importantly, it can precisely control layer number. However, it is dogged by small grain sizes, stoichiometry issues, and creates comparatively low-quality samples.

### 2.4. Atomic layer deposition (ALD)

ALD is a CVD-type growth strategy that incorporates many of the strengths of PLD. While MOCVD and APCVD proceed by exposing the growth substrate to all reactants simultaneously, ALD pulses each reactant sequentially such that only one is ever present in the reaction space at any one time. As with PLD, a pulsing strategy allows for fine control on layer number. Most crucially, the reactions are self-limiting: after each pulse of one precursor there is only a finite number of sites available for the second precursor to react with, preventing uncontrolled epitaxial growth. This also helps to control layer number. Most importantly, ALD TMD growths can occur at very low temperatures—as low as 60 °C. However, ALD growths tend to be quite slow and they demand very expensive equipment.

Tan *et al* [58] grew MoS_2_ monolayer film on the scale of 5 cm on sapphire, at a temperature of 300 °C. They observed that, as with PLD, precursor pulse count corresponded directly to layer number which allowed for selective growth of mono, bi, and trilayer MoS_2_. However, the low growth temperatures inherent to ALD setups lead to poor crystallinity. As a result, annealing at 800 °C was necessary to improve sample quality, confirmed by a much sharper PL emission. However, this annealing lead to the breakup of film, resulting in MoS_2_ islands. Browning *et al* [59] also achieved a 5-cm-scale growth of both monolayer and bilayer MoS_2_, but on Si/SiO_2_. This growth was conducted at 390 °C, at this higher temperature films were of sufficient quality that post-treatment was not necessary [59]. Indeed, they observed no obvious grain boundaries under a scanning electron microscope [59]. With this wafer scale growth, FETs were fabricated out of bilayer MoS_2_, with a channel 100 *μ*m-wide. Transistors constructed over such a large scale would normally perform poorly due to material inhomogeneity over length scales longer than a flake, but even so mobility was around 1 cm^2^ Vs^−1^ [59]. While not as high as FETs with shorter channel length, this is an excellent demonstration of the new scales of electronics permitted by film growths. The growths mentioned thus far utilized inorganic precursors: MoCl_5_ and H_2_S. Jang *et al* [60] used an organic Mo precursor, which enabled further lowering of the growth temperature to 200 °C. This growth rivaled MOCVD efforts, with a monolayer film 10 centimeters in diameter achieved on a sapphire substrate. The sequential depositing strategy of ALD helped avoid a major pitfall of MOCVD: Jang *et al* [60] observed little carbon contamination in their final sample. While this low temperature growth lead to domains on the scale of nanometers, this may be an acceptable price to pay [60] Some applications may demand growing material directly on substrates that are either temperature sensitive themselves (such as flexible substrates) or on substrates with temperature sensitive components upon them (such as photoresists). Low temperature growths, while suffering in crystallinity, are essential for realizing these possibilities. Pursuing this, Jurca *et al* [61] used an organic Mo precursor to synthesize monolayer MoS_2_ on the millimeter scale on Si/SiO_2_ at 60 °C. Taking advantage of this incredibly low growth temperature, Jurca *et al* lithographically pre-patterned the growth substrate to shape the resultant growth [61] While crystallinity was poor, post-annealing improved structural quality [61]. ALD TMD growths are relatively novel compared to more traditional CVD growth strategies, so there is still much to be done in terms of optimization.

Yeo *et al* [62] deposited a 1-cm-scale few-layer WS_2_ on Si/SiO_2_ using an organic tungsten precursor at 350 °C, with layer number varying with pulse number as with MoS_2_. Other efforts have been made to deposit tungsten precursor directly on Si/SiO_2_ via ALD and then sulfurize to grow WS_2_, but we deem these efforts to fall outside the scope of true ALD growths because of the lack of sequential pulsing. MoSe_2_ ALD efforts have proceeded likewise, with ALD deposition of Mo precursor followed by selenization to achieve multilayer centimeter-scale growths on Si/SiO_2_. WSe_2_ has proven much more promising. Browning *et al* [63] achieved 5-cm-scale growths of few-layer WSe_2_ on Si/SiO_2_ at 390 °C using a one-step ALD process in the style of previously mentioned MoS_2_ growths. Mobilities were comparable to CVD growth few-layer WSe_2_ [63].

ALD offers immense promise to deliver large area, layer-controlled growths of various TMDs at temperatures low enough to enable novel substrate modifications. However, the technology must be developed further to enable this.

### 2.5. Other methods

For completeness, we will address a few other growth methods that have been used in TMD synthesis. One technique that has gained recent attention is plasma enhanced CVD (PECVD). This process involves depositing metal precursor on the substrate via e-beam, and then reacting it with chalcogen precursors in the plasma phase [64]. The chalcogen precursor is typically ionized with a current, the energy of the plasma facilitates the TMD synthesis reaction. There is a general tradeoff between growth temperature and growth quality across various synthesis strategies. Use of plasma addresses this, as it provides the energy necessary for the TMD to form properly without necessitating high growth temperatures. Anh *et al* achieved a film growth of few layer MoS_2_ 4 inches in diameter on a flexible plastic substrate at 150 °C. Hall measurements taken at 50 points on the wafer-scale sample revealed an average charge mobility of 2.014 cm^2^ V^−1^ s^−1^ [64]. Increasing the growth temperature to 300 °C, which was still low enough for the use of a flexible substrate, improved the mobility to 3.710 cm^2^ V^−1^ s^−1^ [64]. The domains for the 150 °C and 300 °C were respectively on average 5 nm and 7 nm in diameter; the temperature-crystal quality tradeoff is still present [64]. The samples were 5–6 layers thick on average [64]. However, the group noted that the plasma in the growth chamber tended to create surface defects on the synthesized material, necessitating further optimization to improve sample quality [64]. Kim *et al* optimized the technique further to allow atmospheric pressure PECVD growths of MoS_2_ and WS_2_ [65]. Growths were conducted at temperatures as low as 100 °C, yielding few layer 4-inch diameter growths of both TMDs on silicon as well as on flexible substrates [65]. While domain sizes/mobilities were not measured, both TMDs produced large and fast photocurrent responses to incident laser excitation; these samples were of sufficient quality for optoelectronic applications [65]. PECVD technology to our knowledge has not yielded large area monolayer TMD growth (perhaps because of the plasma-induced surface defects making a single continuous layer currently infeasible). In addition, we cannot identify PECVD growths of selenide TMDs. A related technique, plasma enhanced ALD (PEALD) has also been employed for the growth of MoS_2_. In this process, a plasma-exposure step was introduced between the precursor pulse cycles; with the plasma serving to enhance out-of-plane growth [66]. As with PECVD, this produced multilayer films on the scale of 4 inches at temperatures low enough for flexible substrates, but small domain size was still an issue [66]. Plasma enhanced CVD and ALD technology is promising for its ability to synthesize large area homogenous TMDs at low temperatures, but the technology needs further development in areas such as layer number control and surface defect management in order to reach its full potential.

Molecular beam epitaxy (MBE) is another oft-used method for TMD synthesis, and so worth mentioning in this review. While known for producing samples of exceptional quality, MBE is currently incapable of producing truly large-area TMD growths. Modern MBE growths of TMDs such as MoS_2_, MoSe_2_ and WSe_2_ are restricted to individual flakes with diameters less than a micrometer, a far cry from the ten-centimeter order films produced by other methods [67–69]. As a result, MBE is not currently a promising route to large area TMD synthesis.

## 3. Applications of TMD films

The extraordinary electronic, optical, and mechanical properties of TMDs grant them wide-ranging applications. Chronicling the full scope of these applications is beyond the scope of this article, such detail may be found in other reviews [1, 10, 22–25, 70]. Instead, we will focus on how the specific realization of film growth will garner new applications for TMDs. Graphene provides a compelling analogy once again. The unique applications of graphene films (compared to graphene flakes) anticipate the promises of TMD films. For each application, we may examine how using a TMD film in place of graphene results in advancement. We also consider how existing uses of TMD flakes may be improved significantly with films. The viability of all TMD applications is directly related to the scale and quality of the material being utilized, we therefore give consideration to how better growths lead to optimal performance.

### 3.1. Transistors

Transistors are the basis of modern electronics. However, the scaling limits of silicon threaten to slow current rates of miniaturization progress (enabling denser packing and more computing power). When silicon transistors are made too small, tunneling currents appear which ruin device performance [22]. This issue is not faced by 2D transistors as the low dimensionality of these systems confines electrons, preventing leakage current [22]. TMDs are a natural candidate for the fabrication of transistors and particularly of thin film transistors (TFT), which are extensively used in liquid crystal displays and light emitting diodes. TMDs have sizable band gaps and impressive carrier mobilities that allow for high on/off ratios and fast switching speed, which are the most desirable features of TFTs [71]. Inherent n-typing and p-typing, given the material, enables digital logic circuitry in limited cases. Their thinness means they are very exposed and thus responsive to their environment, priming them for sensing applications and data storage/retrieval [24, 72–74].

Groups have successfully synthesized nanoscale FETs from monolayer MoS_2_, WS_2_, MoSe_2_, and WSe_2_. The exact details of these devices’ performances may be found in other reviews, but in general mobilities are on the order of 10 cm^2^ Vs^−1^, with high on/off ratios, and high on-state current in comparison to conventional Si transistors [75]. In particular, high performance TFTs based on CVD-grown MoS_2_ have been fabricated by many groups. These transistors meet industry standards for use in low-power applications [22]. Interestingly, multilayer TMD based FETs may have properties superior to monolayer ones. Compared to monolayer, few-layer TMDs have a higher density of states, increasing the mobility of the resultant transistors to the order of 100 cm^2^ V^−1^ s^−1^ [76]. Of course, increasing layer number serves to progressively diminish the benefits of low dimensionality.

**Figure 4.**
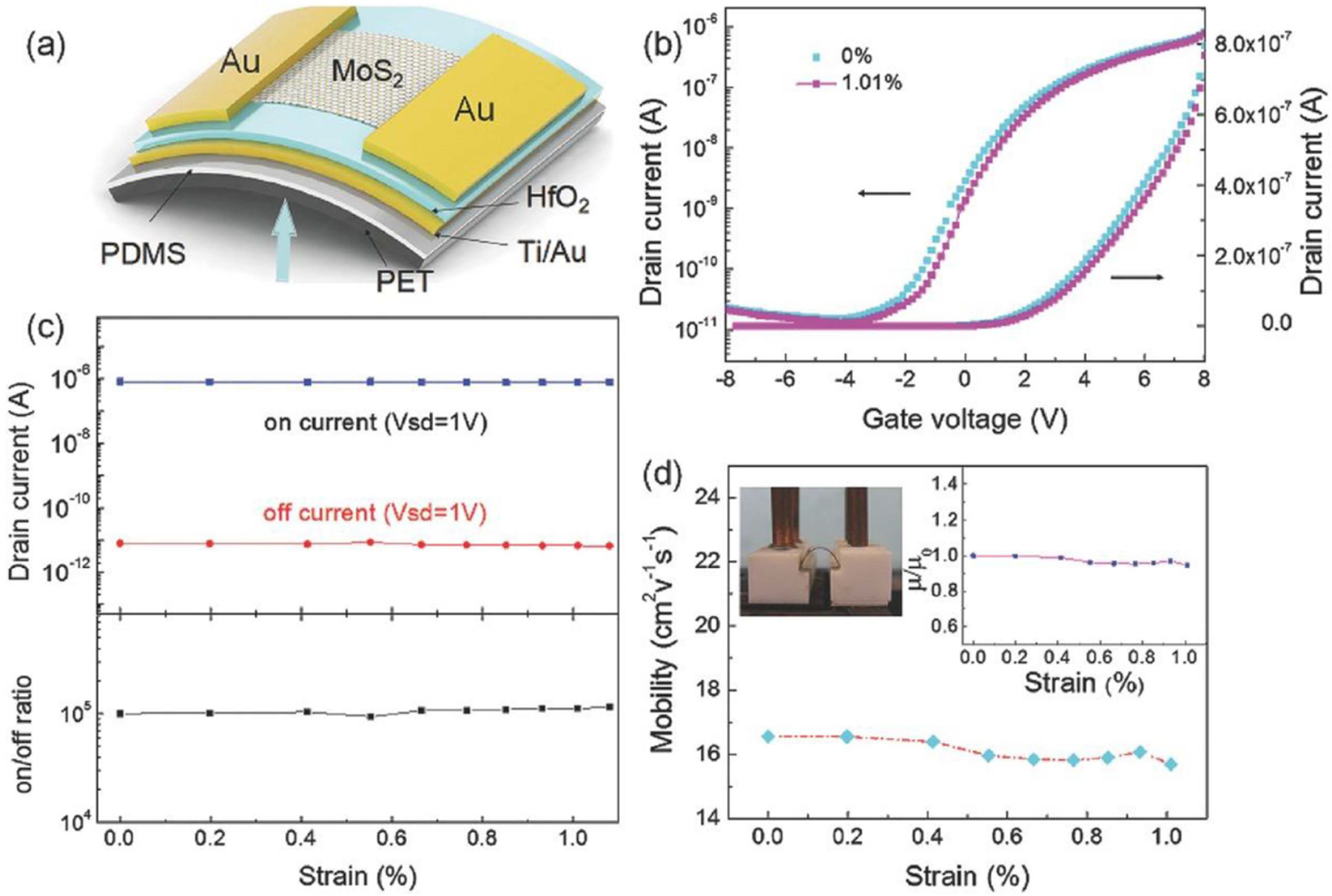
The mechanical flexibility of MoS_2_ FET on PET substrate. (a) The schematic image of the bendable device. The arrow direction shows the strain applied orientation. (b) The transfer characteristics of the device before and after ≈ 1% strain added. (c) Both on and off currents has no obvious change with various strain added, and the on/off ratio over 10^5^ is rarely changed (bias voltage = 1 V). (d) The dependence of the carrier mobility on the strain. The up-left inset is the optical image of the device under strain ≈ 1% and the up-right one is the normalized mobility rarely varied with the strain change. Panels adapted with permission from [11]. Copyright Advanced Electronic Materials 2016.

Large-area synthesis is naturally essential for TMD-based electronic architectures to become commercially relevant, given the sheer scale of the electronics industry. Material uniformity is especially crucial. To compete with silicon architectures, TMD transistors must exploit low dimensionality to be miniaturized further than any 3D-material-based transistor permits. At this scale, individual defects in the lattice will be proportionally catastrophic, given the low number of atoms in the channel to begin with. Selective layer control in growth would enable further tuning of device properties. In addition, the mechanical robustness of TMD makes these transistors prime candidates for flexible electronics, a major trend in modern technology. Graphene, with comparable mechanical properties, has already found use in flexible electronic and optoelectronic devices [77]. Zhao *et al* [11] grew centimeter-scale films of monolayer MoS_2_ with APCVD, which were then transferred to a flexible substrate and patterned into transistor arrays. Devices had mobilities on the order of 10 cm^2^ Vs^−1^ and on/off ratios on the order of 10^5^. These values were essentially unchanged upon 1% uniaxial strain, making these films potentially suitable for applications ranging from curved displays to wearable electronics (figure 4). TFTs fabricated from CVD-grown monolayer flexible MoS_2_ have been known to be electrically stable after thousands of cycles of bending [78].

Once transistors are fabricated, their applications are as broad as the scope of the entire electronics industry. To describe every such application would be impossible; rather, this review concerns itself with the specific benefits of large scale TMD transistor synthesis. Once these benefits are realized, the possibilities are immense.

### 3.2. Optoelectronics

In their monolayer form, TMDs have direct band gaps with energies corresponding to the visible spectrum, as well as large absorption coefficients. This makes them especially suited to use as phototransistors and photodetectors. Such photodevices have applications ranging from imaging to solar cells to spectroscopy [71]. Silicon is often used for these purposes, but it is inefficient due to having an indirect band gap. As with TMD transistors, TMD based optoelectronic devices depend upon the purity and scale of their constituent materials to be effective. Light-harvesting applications naturally demand large areas to capture as many incident photons as possible. In comparison to other TMD efforts, MoS_2_ does not dominate the literature in this subfield. Compared to other TMDs, MoS_2_ has lower absorption and emission efficiency. It is also less thermally stable, which makes it less ideal for applications involving constant optical bombardment. High performance photodetectors have been variously fabricated from MoS_2_, WS_2_, MoSe_2_, and WSe_2_ [3, 6–8, 43, 71, 79–82]. Compared to conventional photodetectors, these devices are distinguished by being especially absorptive, which may be enhanced even further by surface functionalization. Bilayer devices show even higher absorptivity, but layering sacrifices the directness of the band gap [9]. In addition, these devices yield an especially low amount of noise compared to conventional silicon devices, making them suitable for applications that demand precision. Noise has been a consistent weakness for graphene-based photodetectors; the lack of a bandgap leads to much off-state current [77]. However, most of these efforts have focused on flake-based photodevices.

Lan *et al* [43] synthesized centimeter-scale WS_2_ via APCVD as a basis for an array of photodetectors. This work demonstrates the unique promise of film growths: the channels constructed were hundreds of microns long, much longer than the diameter of a typical WS_2_ flake. Flake growths necessary constrain the scale of devices, which limits their applications. Large-scale patterning was also possible; flake-based detector studies rely on ‘bringing the device to the flake’, conducting the arduous process of finding suitable flakes and carefully fabricating devices on them one by one. This inefficient process is a massive barrier to scalability, while film growths like this simply allow patterning entire wafers with a template. Unfortunately, the mobilities of these fabricated devices were quite low, around 1 cm^2^ Vs^−1^, and the on/off photocurrent ratio was weak, around 1.5. However, response time was impressive, on the order of a few milliseconds. When placed in vacuum, the on/off ratio improved but the response time worsened [43]. Further work has been performed on films of WS_2_ and other TMDs, resulting in the fabrication of photodetectors with both faster response times and better photoresponsivity. Improving upon this progress, and the progress done with TMD transistors in general, will require higher quality film growths, as well as better engineering of metallic contacts.

### 3.3. Membranes

Thus far, we have covered applications utilizing the inherent electronic and optical properties of TMD films. In addition to these, it is worth considering how the physical structure of TMDs may be used to creative effect.

**Figure 5.**
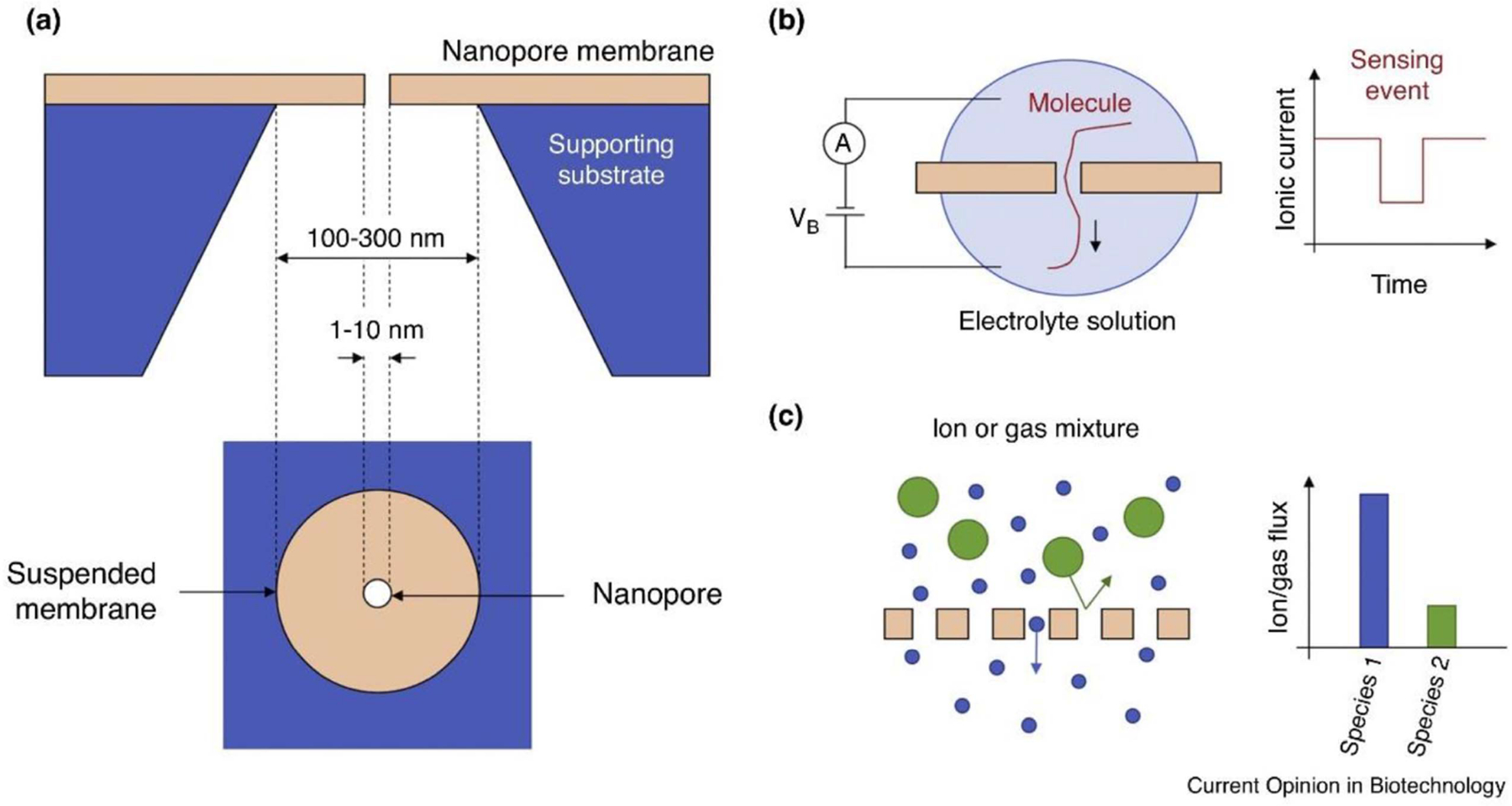
Nanoporous membrane concept (a) schematic of a nanopore channel (b) schematic of ionic current dropping upon translocation, (c) nanopore membrane enabling size-based molecular filtration. Panels adapted with permission from [70]. Copyright Elsevier 2019.

One key example of this is nanoporous membranes, which are described in a recent review by Danda and Drndic (figure 5) [70]. TMD films are naturally impermeable to ions and gas. Holes may be drilled in TMD sheets with electron beams that can be made as small as a few nanometers. These nanopores can serve to selectively filter for molecules based on size, garnering applications in water desalination and gas separation [70]. In addition, TMD nanopores show especial promise for DNA sequencing. Sequencing is performed by immersing a pore in an ionic solution containing DNA, and then driving an ionic current across the pore. When DNA translocates through the pore, it results in a measurable interruption of the ionic current, theoretically allowing identification of individual nucleotides [70, 83]. 2D materials allow for especially high-resolution on the DNA because their thickness is commensurate with that of a single nucleotide of DNA. At any moment, one may expect around one nucleotide to occupy the pore. Compared to graphene, TMD nanopores have higher ionic permeability, making them especially suited for this application [70]. Film growth allows for nanoporous membranes, TMD sheets perforated with nanopores. This would allow for high throughput filtration of fluids or gas in a way that single-flake individual nanopores cannot enable [70]. Das *et al* [84] achieved centimeter scale synthesis of MoS_2_ with layer number averaging 6–7, with monolayer regions surrounded by few-layer regions. Using a chemical etchant, pores formed predominantly in monolayer areas with coverage around 10%. Unlike with electronic and optical applications, random multilayer regions proved useful: providing structural support for the lattice.

TMD membranes have also attracted interest as media for energy storage. Supercapacitors, capacitors with capacitance significantly exceeding that of traditional electrolytic capacitors, have found extensive applications in contexts that require rapid delivery or absorption of large amounts of charge such as regenerative automobile breaking or computational memory backup [85]. Capacitance scales with surface area; 2D materials are prime candidates for supercapacitor materials because of their large surface area to volume ratio. In addition, their mechanical flexibility makes such supercapacitors relevant to wearable electronics. While extensive work has been done on graphene-based supercapacitors, their energy densities are significantly inferior to that of conventional lithium ion batteries. TMDs show more promise due to their more complicated structural chemistry. The three-atom-thick structure of a TMD monolayer allows for charge storage via ionic intercalation, and the variable oxidation states of the transition metal atoms allow for electrostatic charge storage via the formation of an electrochemical double layer [85]. Since area is the signature benefit of 2D material supercapacitors, large-area film growths are naturally essential. Devices synthesized from MoS_2_ films have been reported to have superior performance to conventional carbon-based supercapacitors, with specific capacitance of 100 F g^−1^ [86]. These devices are on the millimeter scale, flakes are clearly incompatible with this approach. WS_2_ based devices achieved around twice the specific capacitance of MoS_2_, but proved to be less durable under cycling [10, 87]. Selenide TMDs, with their superior conductivity, perform even better. MoSe_2_ based supercapacitor electrodes achieved around the same level of capacitance as WS_2_ ones, but with much higher durability [88]. A vast number of approaches have been tried to optimize these devices, including chemical modification and physical restructuring, but these are beyond the scope of this review and may be found in other articles [10, 12, 85–88].

## 4. Conclusion

The unique electrical, optical, and structural properties of TMDs make them essential to the next generation of technology. While much attention has been given to these materials as a whole, there has been little focus on the specific importance of growing them in large areas with pristine quality. In this review, we highlighted the ongoing challenges of synthesizing these materials. We observed some major strategies used by multiple groups: MOCVD, APCVD, PLD, and ALD. MOCVD growths have the benefit of readily synthesizing very large films, up to 10 centimeters in diameter. However, these growths can be slow, use dangerous ingredients, and yield samples of low structural quality. APCVD growths are notable for producing high-quality samples with large domain sizes and using relatively safe and cheaply accessible reaction conditions. Even so, these growths cannot reach the same scale as MOCVD growths. PLD growths are quick, can achieve fine control on layer count, and occur at relatively low temperatures. Unfortunately, the resultant products are generally smaller in scale and often have poor stoichiometry which results in mediocre device performance. ALD growths, in addition to achieving impressive scale and quality, can occur at temperatures low enough to permit the use of more sensitive substrates. However, these growths are quite slow and expensive, and often require post-treatment. Plasma-enhanced synthesis methods allow for low-temperature wafer scale growths without post-treatment, but they suffer from surface defects and small domain sizes. Overall, we note broad trends towards larger areas of growth, higher quality of grown material (i.e. larger domain sizes and improved optical/electronic properties) improved tunability of layer number, and increased throughput.

We also noted the unique practical benefits of achieving centimeter-scale few-layer TMD film growths. The scaling limits of silicon demand new transistor architectures, and TMDs have proven themselves as prime candidates as a consequence of their low dimensionality. With large-area growths, groups have patterned arrays of transistors that meet current industrial standards. TMDs are distinct among 2D materials for having a direct band gap, and this makes them excellent at harvesting light. Large-scale growths have enabled the fabrication of arrays of photodetectors, devices that must necessarily be scaled up to capture enough light to be useful. Lastly, large sheets of few-layer TMDs enable applications that exploit their large surface area to volume ratio, ranging from energy storage to DNA sequencing.

There is still much room for improvement. The growth strategies mentioned above struggle to optimize scale, quality, and cost of synthesis all at once, with each method having its own strengths and weaknesses. With the potential of the aforementioned applications, these three main parameters must be advanced further, especially for TMDs besides MoS_2_. Another major concern is the repeatability of these methods. In general, studies in this field do not routinely provide a quantitative assessment of how reproducible their synthesis methods are. Because reliability is a necessary prerequisite for the implementation of a growth strategy, it is important to know what percent of the time a given method produces a satisfactory sample. This represents an important challenge for the future. Applications of these films are also still nascent; many fabricated devices do not exploit the inherent potential of TMDs sufficiently to significantly outcompete conventional architectures. Furthering the synthesis and application of few-layer TMD films will have great implications for the future of technology.
